# Anti‐platelet factor 4 immunoglobulin G levels in vaccine‐induced immune thrombocytopenia and thrombosis: Persistent positivity through 7 months

**DOI:** 10.1002/rth2.12707

**Published:** 2022-05-04

**Authors:** Samantha J. Montague, Christopher W. Smith, Clare S. Lodwick, Charlotte Stoneley, Matthew Roberts, Gillian C. Lowe, William A. Lester, Steve P. Watson, Phillip L. R. Nicolson

**Affiliations:** ^1^ 1724 Institute of Cardiovascular Sciences College of Medical and Dental Sciences University of Birmingham Birmingham UK; ^2^ Department of Haematology Worcester Acute Hospitals NHS Trust Worcester UK; ^3^ 1732 Department of Blood Sciences University Hospitals Birmingham NHS Foundation Trust Birmingham UK; ^4^ 1732 Comprehensive Care Haemophilia Centre University Hospitals Birmingham NHS Foundation Trust Birmingham UK

**Keywords:** anti‐PF4 antibodies, AZD1222 vaccination, FcγRIIA, thrombosis, VITT

## Abstract

**Background:**

Anti‐platelet factor 4 (PF4) antibodies that activate platelets via FcγRIIA drive the pathophysiology of vaccine‐induced immune thrombocytopenia and thrombosis (VITT). Evolution of these antibodies and their ability to activate platelets after initial treatment remains unknown.

**Objectives:**

To assess how clinical and platelet parameters, anti‐PF4 antibody levels, and patient serum reactivity changes during follow‐up after VITT presentation.

**Methods:**

We describe cases of seven discharged VITT patients that were followed from diagnosis up to 280 days (range 199–280) after vaccination. We measured anti‐PF4 antibodies and PF4 levels in patient serum during follow‐up and tested the ability of patient serum to activate healthy donor platelets and patient platelets over time.

**Results:**

Anti‐PF4 immunoglobulin G antibody levels are very high at diagnosis (0.9–2.6 OD) and remain relatively high (>1.0 OD) in all patients, except one treated with rituximab, at 7 months post vaccination. All patients were on direct oral anticoagulants throughout follow‐up and no patients had recurrent thrombosis. Patients’ platelets during follow‐up have normal FcγRIIA levels and responsiveness to platelet agonists. Patient diagnostic serum strongly activated control platelets, either alone or with PF4. Most follow‐up serum alone was weaker at stimulating donor and patient platelets. However, follow‐up serum beyond 150 days still strongly activated platelets with PF4 addition in three patients. Patient serum PF4 levels were lower than controls at diagnosis but returned within normal range by day 50.

**Conclusions:**

Explanations for reduced platelet activation during follow‐up, despite similar total anti‐PF4 antibody levels, remains unclear. Clinical implications of persistent anti‐PF4 antibodies in VITT require further study.

**Essentials**

- Some COVID‐19 vaccines can cause vaccine‐induced immune thrombocytopenia and thrombosis (VITT).
- We measured lab tests for VITT patients after recovery in affected patients.
- Anti‐Platelet Factor‐4 antibody levels were high after 7‐months but platelet activation reduced.
- Implications of reduced platelet activation despite high anti‐PF4 antibody levels remain unclear.

## 1 INTRODUCTION

Vaccination is the major defense against the SARS‐CoV‐2 pandemic. In April 2021, a new syndrome of vaccine‐induced immune thrombocytopenia and thrombosis (VITT) was described.^1^, ^2^, ^3^ VITT occurs 4 to 30 days following Oxford‐AstraZeneca ChAdOx1 nCoV‐19 (AZD1222) vaccination in 1/50,000 to 1/100,000 people vaccinated in the United Kingdom,^4^ with a potentially lower incidence after Janssen/Johnson & Johnson (Ad26.COV2.S) vaccination reported in the United States.^5^, ^6^ VITT is characterized by thrombocytopenia, high D‐dimer levels, and aggressive thrombosis at unusual venous sites, including cerebral venous sinuses. Arterial thromboses have also been described.^7^, ^8^ Serum from patients with VITT has been shown to activate platelets via the FcγRIIA receptor.^1^, ^2^, ^9^, ^10^ Patients with VITT have very high levels of antibodies to platelet factor 4 (PF4)^3^ that bind with high affinity to a single site on PF4.^9^

Vaccine‐induced immune thrombocytopenia and thrombosis treatment modalities have evolved rapidly and include non‐heparin anticoagulation, corticosteroids, intravenous immunoglobulin (IVIg), thrombectomy, and plasma exchange.^7^, ^11^ Rituximab has also been used in patients who are nonresponsive to first‐line therapies.^7^ Despite treatment, mortality remains around 20% to 50% depending on presence of intracerebral bleeding, geographical region, and ability to deliver early treatment.^4^, ^12^, ^13^ Those that respond do so quickly and are discharged on non‐heparin anticoagulation.

Here, we describe the clinical course, laboratory results, anti‐PF4 IgG, PF4 serum levels, and the ability of serum to activate healthy donor and patient platelets in seven patients with VITT who survived their initial presentation and were followed for up to 280 days after vaccination with AZD1222.

## 2 METHODS

### 2.1 Patients and ethical approval

All patients presenting with VITT at our center who survived to discharge were recruited. Blood was collected at diagnosis and each time the patients arrived for clinical follow‐up. Informed consent was provided by the patients or next of kin in those who lacked capacity. Patient blood collection was approved under research ethics 15/NW/0079, amendment 3. Ethical approval for collecting blood from healthy volunteers was granted by Birmingham University Internal Ethical Review Committee (ERN_11‐0175) and for AZD1222 vaccinated controls from the COCO study was approved under research ethics 20/HRA/1817. All AZD1222‐vaccinated control samples were from 4 to 30 days following first dose of vaccine. All studies were performed in line with the Declaration of Helsinki. Full clinical case histories of the seven patients with VITT are reported in Supplementary Materials.

#### 2.1.1 Materials

PF4 was from Chromatec GmbH (Greifswald, Germany), collagen‐related‐peptide was from CambCol Ltd (Ely, UK), FITC‐conjugated mouse anti‐human CD32a antibody was from BD Pharminogen (Wokingham, UK), FITC‐conjugated immunoglobulin G1 (IgG1) control mouse antibody was from Dako (Santa Clara, CA). Anti‐PF4 IgG ELISA kit was from Immucor (Waukesha, WI). PF4 Human ELISA kit was from Invitrogen (Waltham, MA). All other reagents were from Sigma‐Aldrich (Poole, UK).

### 2.2 PF4 antibody and PF4 serum level measurements

Patient serum samples were collected at diagnosis and multiple follow‐up timepoints. Healthy control serum was taken from multiple donors, including AZD1222‐vaccinated healthy controls (*n* = 4, from the COCO study) and the same‐day controls (*n* = 3, healthy non‐age‐matched controls processed alongside follow‐up serum). Serum was collected following centrifugation (2000 *g*, 10 min, room temperature [RT]) of clotted blood. Anti‐PF4 antibodies were measured using an anti‐PF4/heparin enzymatic immunoassay (EIA, LIFECODES PF4 IgG assay; Immucor GTI Diagnostics) for IgG PF4‐heparin antibodies. Anti‐PF4 antibodies in patient serum (diluted 1:50) from all time points were measured on the same day using a Molecular Devices platelet reader (wavelength 405 nm). Serum PF4 levels were measured using the PF4 human ELISA kit (Invitrogen). Serum was diluted 1:500 in assay buffer and performed according to the manufacturer's protocol. Patient samples were measured in duplicate.

### 2.3 Platelet studies

#### 2.3.1 Preparation

Washed platelets were prepared from citrated whole blood as described.^14^ Briefly, citrated blood was taken from healthy, drug‐free volunteers and mixed (10:1, v/v) with acid citrate dextrose and centrifuged (200 *g*, 20 min, RT) to produce platelet‐rich plasma (PRP). 0.2 μg/ml prostacyclin was added to platelet‐rich plasma before centrifugation (1000 *g*, 10 min, RT). Platelet pellet was then resuspended in modified‐Tyrode‐HEPES buffer, acid citrate dextrose, and 0.2 μg/ml prostacyclin and centrifuged (1000*g*, 10 min, RT). Platelets were resuspended in modified‐Tyrode‐HEPES and rested for 30 min before use.

#### 2.3.2 Aggregation

Light transmission aggregation of patient and control washed platelets (2 × 10^8^/ml) was measured under stirring conditions (1200 rpm) at 37 °C for 30 min following stimulation with collagen‐related‐peptide, thrombin, or serum (14:1, v/v) in the presence of 10 μg/ml PF4 or vehicle. Aggregation data presented as area under the curve calculated with Aggrolink 8 software (Chrono‐log, Havertown, PA).

#### 2.3.3 Platelet FcγRIIa (CD32a) surface measurements

Washed platelets (2 × 10^8^/ml) were stained for 30 min with mouse anti‐human CD32a (FcγRIIA antibody) or FITC control antibody before termination with excess phosphate buffered saline and median fluorescence intensity measured using flow cytometry (BD Accuri C6).

### 2.4 Statistical analysis

All data are presented as mean ± standard deviation (SD) unless stated. Statistical comparisons performed by one‐way ANOVA with Tukey multiple comparisons after normality tests. All analysis performed with GraphPad Prism 9.0 (La Jolla, CA).

## 3 RESULTS AND DISCUSSION

**FIGURE 1:**
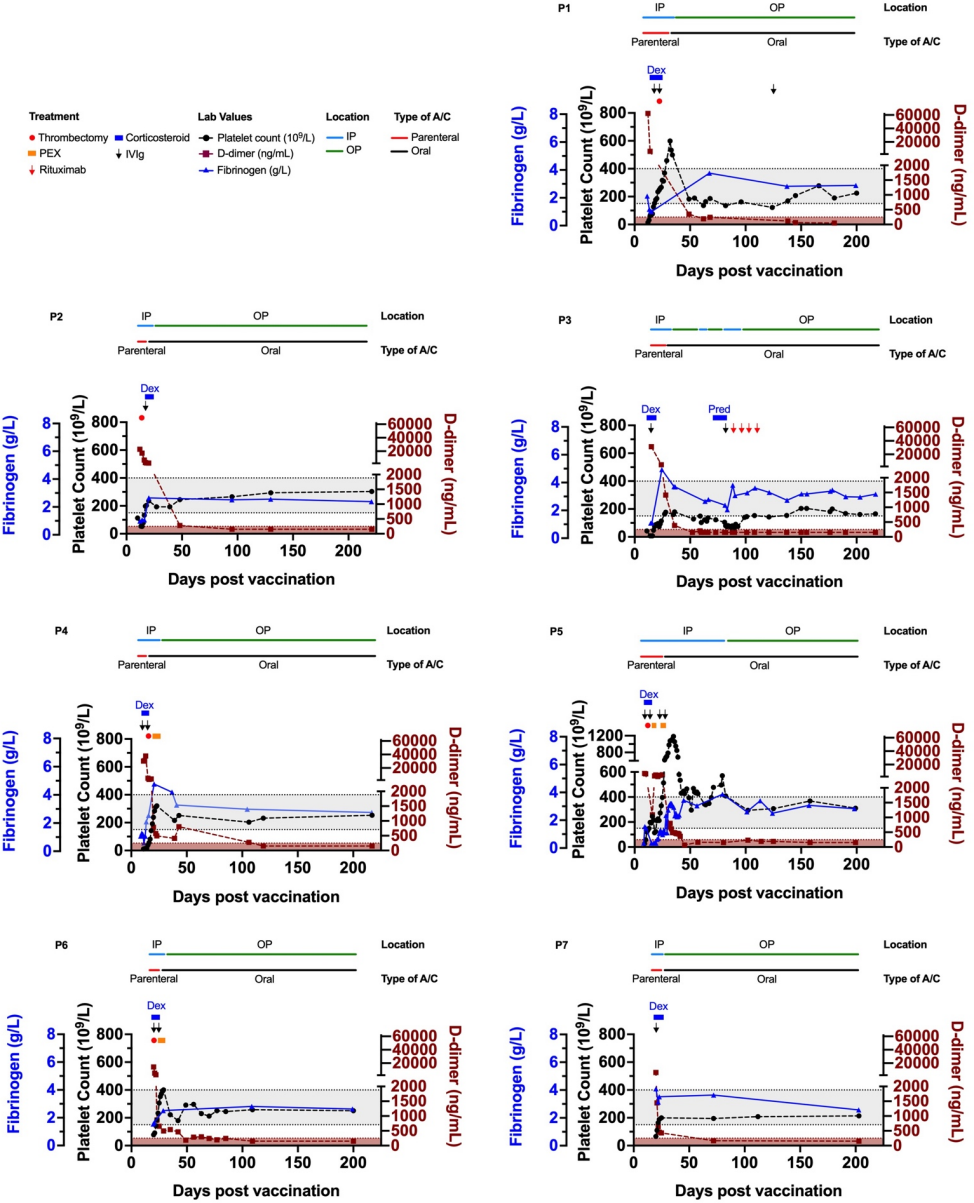
Laboratory parameters of seven patients with VITT 199 to 217 days following vaccination with AZD1222. Gray box denotes normal range for platelet count and fibrinogen, brown box denotes normal range for D‐dimer. A/C, anticoagulation; Dex, dexamethasone; IP, inpatient; IVIg, intravenous immunoglobulin; NR, normal range; OP, outpatient; PEX, plasma exchange; Pred, prednisolone. The patients are identified by their number (P1‐P7). Laboratory parameters and details of clinical information of the patients with VITT can be found in Supplementary Materials

Clinical and laboratory parameters of seven patients with VITT during follow‐up (range 199–217 days) after the first dose of AZD1222 vaccination are shown in Figure 1. All patients were white and were on therapeutic anticoagulation with direct oral anticoagulants (DOACs) throughout follow‐up. Five patients remained in remission with normal platelet counts and minimal symptoms. Two patients (P1 and P3) showed signs of relapse with mild thrombocytopenia and headache but did not develop recurrent thrombosis. Both were treated with IVIg; P3 was subsequently treated with rituximab. D‐dimers and fibrinogen levels were normal in all patients after discharge. This may be due to ongoing anticoagulation. The recurrence of thrombocytopenia despite treatment with DOACs raises the possibility that, similar to patients with triple‐positive antiphospholipid syndrome, vitamin K antagonists might have a role in VITT treatment.^15^

**FIGURE 2:**
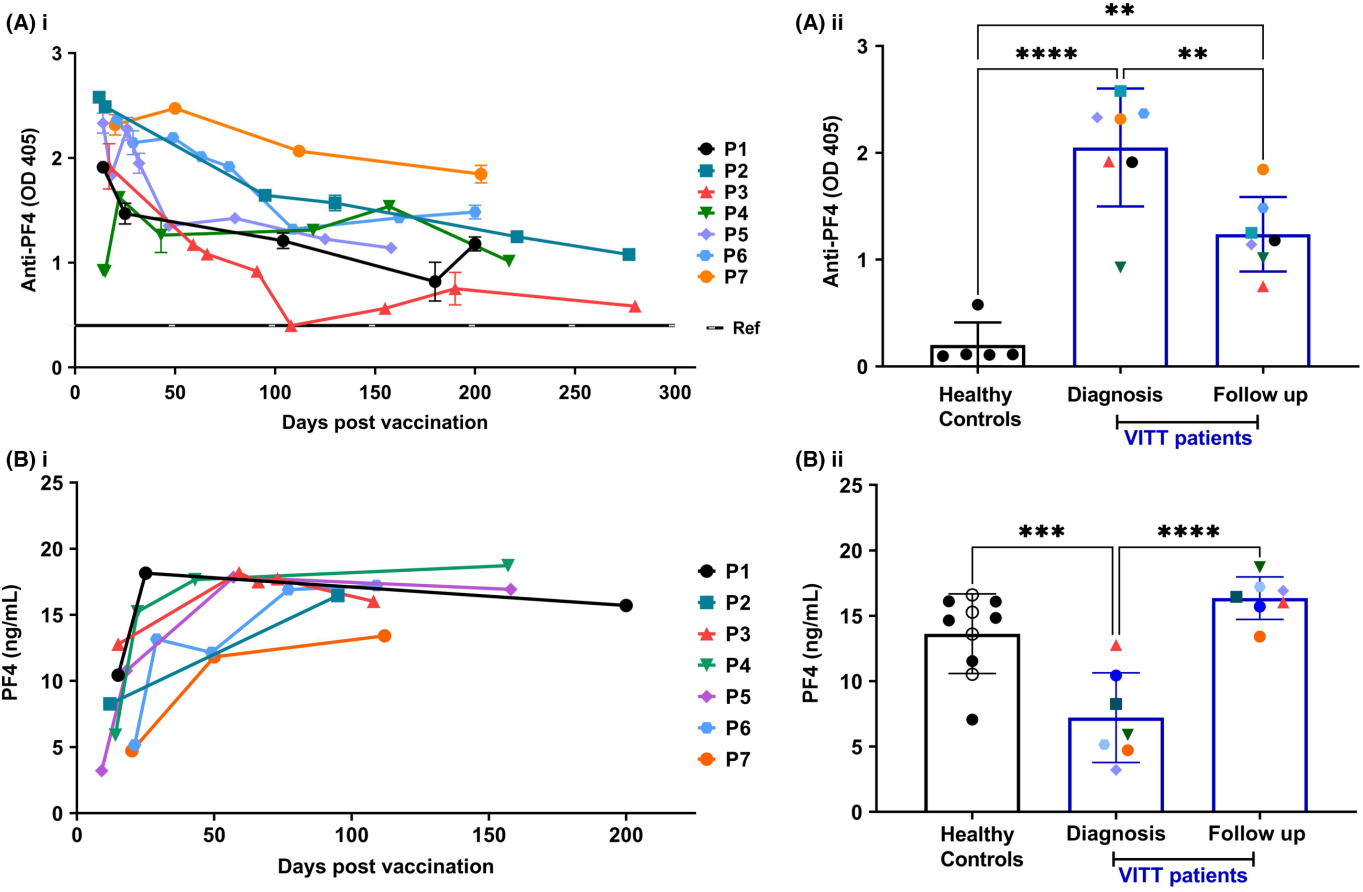
Levels of anti‐PF4 IgG and PF4 level's in the serum of patients with VITT during follow‐up. (Ai) Serum anti‐PF4 levels of VITT patients (*n* = 7) at diagnosis and during follow‐up timepoints after vaccination. Samples measured in duplicate, mean ± SEM. OD 405, optical density at 405 nm. Ref, clinical control reference range <0.4 O.D. (Aii) Patient serum anti‐PF4 IgG levels at diagnosis and later follow‐up timepoint (nearest to 200 days) (*n* = 7) compared with healthy control serum levels (*n* = 5, including serum from the three responsive donors). Individual patient symbol corresponds to patient labels in (i). Results reported as mean ± SD. One‐way ANOVA with Tukey's multiple comparison test, **p < 0.01, ****p < 0.0001. (Bi) Serum PF4 levels of all patients with VITT (*n* = 7) at diagnosis and follow‐up timepoints after vaccination. (Bii) Patient serum PF4 levels at diagnosis and latest follow‐up timepoint (*n* = 7) compared with healthy controls (*n* = 10), including AZD1222‐vaccinated individuals (*n* = 4, open circle). Individual patient symbol corresponds to patient labels in (i). Results reported as mean ± SD. One‐way ANOVA with Tukey multiple comparison test, ***p = 0.0005, ****p < 0.0001

Patient serum anti‐PF4 IgG levels were measured at diagnosis and longitudinally during follow‐up and compared with healthy reference levels (ref: <0.4 OD) (Figure 2Ai). Anti‐PF4 IgG levels in patients with VITT were high at diagnosis (<30 days after vaccination). In most patients, anti‐PF4 levels were initially sustained and only showed a significant reduction by day 200 (**p < 0.01, Figure 2Aii). The rituximab‐treated patient (P3) showed a strong decline in anti‐PF4 IgG levels up to 100 days after vaccination, but anti‐PF4 IgG levels did rise during the next 100 days. Despite immunosuppressive treatment with corticosteroids, anti‐PF4 IgG levels still remained significantly higher in all patients with VITT compared with healthy controls (**p < 0.01, Figure 2Aii) and above clinical reference level (>0.4 OD) for > 200 days following AZD1222 vaccination.

Because PF4 can potentiate platelet activation *in vitro* in patients with VITT, we measured serum PF4 levels in patients at diagnosis and over time to assess if high PF4 levels were present at the time of diagnosis or potentially contributed to relapse (Figure 2Bi). PF4 serum levels at diagnosis were reduced by approximately 50% compared with follow‐up levels and healthy controls (*p* < 0.0001 and *p* = 0.0005, respectively; Figure 2Bii). This may reflect the thrombocytopenia at diagnosis because platelets are the source of PF4, or possibly consumption by binding to high‐affinity circulating antibodies forming the pathogenic immune complexes. PF4 levels of healthy controls vaccinated with AZD1222 (open circle) are similar to other controls (Figure 2Bii).

**FIGURE 3:**
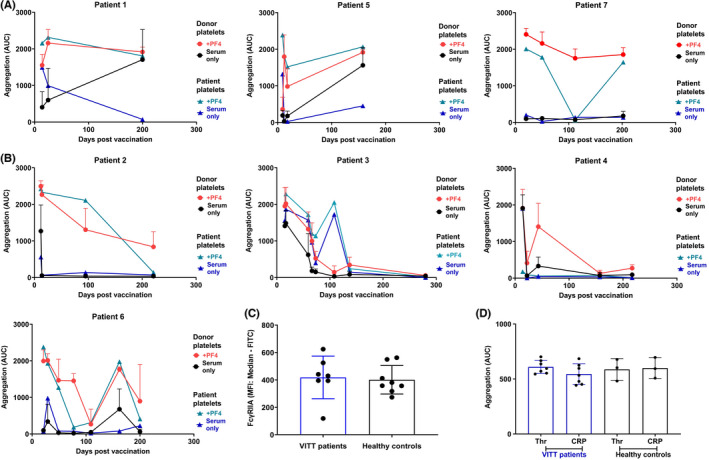
Measurement of patient serum‐mediated platelet aggregation in controls and patient platelets, patient platelet responsiveness, and PF4 serum levels. (A) Serum for three VITT patients (P1, P5, P7) activates platelets during follow‐up. Aggregation (area under the curve [AUC]) of healthy “responsive” donor platelets (mean + SD, *n* = 3) and VITT patient platelets (2 × 10^8^/ml) with diagnostic and follow‐up patient serum (14:1 v:v) in the absence and presence of 10 µg/ml PF4. (B) Decline in platelet aggregation with follow‐up serum for four VITT patients (P2, P3, P4, P6) with donor and patient platelets. (C) Median fluorescence intensity of CD32a (FcγRIIA)‐positive washed platelets (2 × 10^7^/ml) (*n* = 8) healthy controls (black), including the three responsive controls, and *n* = 7 VITT patients (P1; day 200, P2; day 221, P3; day 280, P4; day 157, P5; day 158, P6; day 109 and P7; day 112 after vaccination, VITT patient results shown in blue). Results shown as mean ± SD. (D) Aggregation (AUC) of patient (*n* = 7) and healthy “responsive” control platelets (*n* = 3) with 0.1 U/ml thrombin or 1 μg/ml collagen‐related‐peptide for 8 min. VITT patient results shown in blue

We next compared the ability of diagnostic and follow‐up serum to activate platelets in the absence or presence of PF4. Patient serum taken at diagnosis or during follow‐up was tested on washed platelets from three healthy donors who were previously found to be responsive to VITT serum.^10^ The same three donors were used for all patient serum at all timepoints. Serum taken at or soon after diagnosis from all seven patients induced powerful aggregation, which in three cases (P2, P3, P4) did not require addition of PF4 (Figure 3A,B). Immediately following initial treatment for VITT (steroids, IVIg, and plasma exchange), serum from P2, P3, and P4 no longer/weakly activated healthy control platelets without additional PF4, with the PF4 enhancement also diminishing over time (Figure 3B). We previously showed IVIg and plasma exchange reduced patient serum‐mediated platelet activation.^10^ However, it is interesting that in some patients, exogenous PF4 rescued this. Aggregation to P3, P6, and P7 serum in the presence of PF4 also decreased over time, although mild and robust aggregation could be observed to P6 serum at day 157 and P7 serum at day 112, respectively (Figure 3). In stark contrast, the latest follow‐up serum from P1 (200 days) and P5 (158 days) stimulated full aggregation of platelets in the absence of PF4 and full aggregation was seen in the presence of PF4 to all their other follow‐up samples (Figure 3A). The variation in response over time could relate to changes in anti‐PF4 levels (e.g., a small increase was observed at day 200 with P1, although no change was seen with P5) or be indicative of changes in reactivity of the anti‐PF4 antibodies.

We were able to obtain washed platelets from all seven VITT patients at their latest follow‐up timepoint. We investigated the effect of diagnostic and follow‐up serum from each patient on their own platelets. Each patient's own diagnostic serum caused robust aggregation either in the absence (P1, P3, P4, P5) or presence of PF4 (P2, P6, P7) (Figure 3A,B). The latest follow‐up serum from five patients (P2, P3, P4, P6, P7), however, was unable to induce activation of their own platelets, even in the presence of PF4. In the remaining patients (P1 and P5), follow‐up serum mediated robust activation of patient platelets in the presence of PF4 (Figure 3A). The greater reactivity to P1 and P5 serum is in line with the results on the donor platelets, although in this case, the last follow‐up samples did not require PF4. The lack of clinical change in the patients despite the high platelet reactivity may reflect ongoing use of anticoagulation. Interestingly, serum from P3 at day 108 was able to induce strong platelet aggregation of the patient's platelets even in absence of PF4, which was not observed with donor platelets, nor was it associated with any increase in anti‐PF4 levels at that time. This was at the same time as their clinical relapse and before their subsequent treatment with rituximab.

We wanted to assess platelet characteristics and function of patient platelets. FcγRIIA (CD32a) levels were measured on all patients’ platelets at latest follow‐up timepoint, and most were found to be similar levels to healthy controls (Figure 3C). P3’s platelet FcγRIIA levels were low compared with controls and other VITT patients. However, the responsiveness of all patients’ platelets to thrombin (0.1 unit/ml) and collagen‐related‐peptide (1 μg/ml), measured at latest follow‐up timepoint, was similar to controls (Figure 3D).

This study shows that although anti‐PF4 IgG antibody levels are high at diagnosis and, although they decline during follow‐up, they still remain above control reference range 7 or more months following AZD1222 vaccination. In our cohort, there is only an average 38% reduction in anti‐PF4 IgG levels (range −10% to 69%) during follow‐up. These patients still do not relapse, however. Two recent studies have also showed a decline in anti‐PF4 IgG levels in VITT patients in a shorter follow‐up period (median 11–12 weeks).^16^, ^17^ Interestingly only 3/35 patients^16^ and 2/6 patients^17^ in these studies had a negative anti‐PF4 IgG result (<0.5 OD) during the shorter follow‐up period. The Schönborn et al. study also showed a reduction in PF4‐dependent platelet activation of donor platelets with patient follow‐up serum, which became negative in 23/35 patients during initial follow‐up.^16^ Here, we expanded on these conclusions with longer follow‐up timepoints and more extensive characterization of patient follow‐up serum‐mediated platelet activation of both donor and patient platelets. We show 6/7 patients still have an anti‐PF4 IgG level of >1.0 OD after a median of 30 weeks and 5/7 and 2/7 patients still have detectable PF4‐dependent and PF4‐independent platelet activation respectively, with serum having the potential to activate patient and donor platelets. We consider this particularly important given that light transmission aggregation is considered a lower sensitivity assay than the gold standard serotonin release assay in a similar condition, heparin‐induced thrombocytopenia (HIT).^18^ Further, we show that serum PF4 levels reduce at VITT presentation, but return to normal over time and that the responsiveness of the patients’ own platelets and FcγRIIA levels are similar to controls. The absence of relapse may be due to ongoing treatment with DOACs. It may also be due to consumption of a subset of high affinity or platelet‐activating anti‐PF4 IgG antibodies that are required to generate immune complexes at initial diagnosis that are no longer present during follow‐up.

One limitation in the present study is the reliance on ELISAs to measure anti‐PF4 IgG antibodies. These do not quantitatively measure the levels of the subset of reactive antibodies or determine whether monoclonal or polyclonal antibodies are key contributors to VITT. Further investigation and development of assays to measure changes in levels of reactive antibody over time is warranted to determine whether high levels of platelet‐activating anti‐PF4 antibodies can persist during follow‐up and lead to potential relapse in VITT patients^19^ or reduce over time similar to the decline in anti‐PF4 antibodies observed with HIT and autoimmune HIT.^20^, ^21^ Furthermore, because the latest follow‐up serum in two patients, P1 and P5, mediated powerful activation of donor and patient platelets, even with declining anti‐PF4 IgG levels, it suggests reliance on one clinical test, either anti‐PF4 IgG ELISA results or platelet function alone is not advisable. Patients with VITT should continue to be maintained on anticoagulation until more is known about the relationship between anti‐PF4 antibody levels, serum ability to activate platelets, or indeed other biomarkers, and risk of relapse.

## RELATIONSHIP DISCLOSURE

Phillip L. R. Nicolson and Steve P. Watson have received research grants from Novartis, Principia and Rigel Pharmaceuticals. Phillip L. R. Nicolson has had honoraria from Bayer, Grifols, Takeda, and Sobi. All other authors report no relevant conflict of interests.

## AUTHORS CONTRIBUTIONS

Phillip L. R. Nicolson recruited patients, collected and analyzed clinical data, designed and performed experiments, and wrote the manuscript. Samantha J. Montague designed and performed experiments, analyzed data, and wrote the manuscript. Christopher W. Smith designed experiments, analyzed data, and wrote the manuscript. Clare S. Lodwick collected clinical data. Charlotte Stoneley and Matthew Roberts performed experiments. Steve P. Watson designed experiments and wrote the manuscript. Gillian C. Lowe and William A. Lester recruited patients and contributed intellectually. All authors approved the final manuscript.

## Supporting information

Supplementary Material

Supplementary Material

## Data Availability

Deidentified participant data and the data dictionary can be provided on request to the corresponding author following publication after signing of a data access agreement.
