# Supplementary material for: Anti‐platelet factor 4 immunoglobulin G levels in vaccine‐induced immune thrombocytopenia and thrombosis: Persistent positivity through 7 months
Source: Res Pract Thromb Haemost. 2022 May 4;6(3):e12707. doi: 10.1002/rth2.12707 (PMC9066380; doi:10.1002/rth2.12707)
Supplement: Supplementary file 1 — Supplementary Material [file RTH2-6-e12707-s001.pdf]

Supplementary material

Anti-platelet factor 4 immunoglobulin G levels in vaccine-induced immune thrombocytopenia and thrombosis: persistent positivity through 7 months

Samantha J. Montague, PhD [1]; Christopher W. Smith, PhD [1]; Clare S. Lodwick, MB ChB [2]; Charlotte Stoneley, MSc [3]; Matthew Roberts, MSc [3]; Gillian C. Lowe, MB BChir PhD [4]; William A. Lester, MB ChB PhD [4]; Steve P. Watson, PhD [1]; Phillip L.R. Nicolson, MB BChir PhD [1,4]

VITT patient case series

The presenting clinical features and laboratory results of 5 of the 7 patients below have been previously reported.[10] In total we recruited 10 VITT patients to our centre, however 3 patients died soon after diagnosis. We were able to follow-up 7 patients with VITT at times where they were reviewed in clinic. A brief summary of these patients is included in Supplementary Table 1.

Patient (P) 1 (who suffered a CVST) was discharged on dabigatran after a 24-day admission. At this point he had a mild headache and some ongoing pyramidal weakness all of which gradually resolved over the subsequent three months. In response to a persistent mild thrombocytopenia (122-135 x10^9/L) and increasing headaches (but with normal D-dimer and fibrinogen levels and without recurrence of CVST) he was retreated with 1 g/kg IVIg on Day 137. After this his platelet count increased to normal and his symptoms ceased (Figure 1). The high PF4 antibody level at diagnosis has remained high during follow up at 200 days post vaccination (1.91 and 1.18 OD respectively) (Figure 2A).

P2 was in hospital for 10 days after a right middle cerebral artery (MCA) infarct. Following discharge on apixaban he has had ongoing mild left sided weakness. This is gradually resolving at 221 days post vaccination. He did not have a headache. His platelet count, D-dimer and fibrinogen have remained normal for the 6 months following discharge (Figure 1).

His PF4 antibody level was very high at diagnosis (2.58 OD) and was still above 1.5 OD after 130 days (Figure 2A).

P3 was discharged on dabigatran 18 days after her CVST. She had ongoing problems with headaches requiring readmission on two occasions (days 65 and 81 following vaccination). Both of these admissions were associated with recurrence of mild thrombocytopenia (67-149 $x10^9$/L) but a normal D-dimer and fibrinogen. During these admissions she started treatment with prednisolone and then subsequently IVIg plus rituximab respectively. Following these further treatments her platelet count normalised, her headache resolved and her PF4 IgG antibody level reduced near to the normal range of <0.4 OD at day 108, although this has risen slightly in the next 100+ days (see Figure 1 and 2A).

P4 suffered a CVST and underwent thrombectomy, IVIg and plasma exchange before being discharged on dabigatran after 11 days. Her headache rapidly resolved and 217 days following discharge she is completely symptom free, has a normal platelet count, D-dimer and fibrinogen (Figure 1). Her follow-up PF4 antibody level has remained high since her diagnosis and continues to be ≥1.0 OD for over 200 days (Figure 2A).

P5 had progressive thrombosis, recurrent thrombocytopenia and ongoing hypofibrinogenaemia 10 days after his initial therapy and required re-treatment. He then developed a ventilator-associated pneumonia (VAP) with resultant thrombocytosis. He was transferred for specialist neurological rehabilitation because of ongoing right sided weakness 73 days after his initial presentation with CVST and was finally discharged home after a further 50 days with good resolution of this weakness. He has ongoing issues with higher executive function. Following successful treatment of his VAP his platelet count, D-dimer and fibrinogen have been normal but his PF4 IgG antibody levels remain persistently >1.0 OD (see Figure 1 and 2A).

A sixth patient (a 47 year old female, P6) who was not included in our previous study developed VITT and suffered a CVST 20 days following her first vaccination with AZD1222. Her presenting PF4 antibody level was 2.37 OD. She was initially treated with fondaparinux,

dexamethasone and IVIg but also required thrombectomy and plasma exchange. She was discharged on dabigatran 9 days after admission. Her PF4 IgG antibody level remained very high for 70 days and has only reduced to 1.48 OD by day 200 post vaccination but her platelet count, fibrinogen and D-dimer remain normal (see Figure 1 and 2A). She has had an ongoing mild headache since discharge.

A seventh patient (a 48 year old female, P7) who was also not included in our previous study, presented with VITT and resultant CVST 20 days following AZD1222. Her PF4 IgG antibody level was 2.32 OD. She was treated with fondaparinux, dexamethasone and IVIg and was discharged on dabigatran 5 days after presentation. Her platelet count, D-dimer and fibrinogen level have remained normal and at latest follow-up (203 days following vaccination) she had no symptoms but still had a high PF4 IgG antibody level of 1.84 OD (see Figure 1 and 2A).

Of note only Patient 1 was treated with heparin-based anticoagulation and this was only for two days immediately after his presentation before it was switched to a non-heparin-based regimen. Otherwise none of the patients received any heparin-based anticoagulation at any point during their treatment.

	Patient 1	Patient 2	Patient 3	Patient 4	Patient 5	Patient 6	Patient 7
**Age**	48	21	46	43	44	47	48
**Sex**	Male	Male	Female	Female	Male	Female	Female
**Site of thrombosis**	CVST	Ischaemic stroke	CVST	CVST	CVST	CVST	CVST
**Number of days post vaccine at presentation**	14	10	14	11	9	20	20
**Platelet count at presentation ($x10^9$/L)** normal range 150 - 450	16	113	7	11	35	78	66
**D-dimer at presentation (ng/mL)** normal range 0 - 250	62342	22903	31301	30324	6807	14274	6035
**Fibrinogen at presentation (g/L)** normal range 1.5 - 4	1·2	0·98	1·1	1.07	<0·35	1.54	4.09
**Anti-PF4 IgG antibody level at presentation (Optical density)** normal range 0.01 - 0.4	1.91	2.58	1.92	0.92	2.33	2.37	2.32
**Anti-PF4 IgG antibody level at latest follow-up (Optical density)** Normal range 0.01 - 0.4	1.18	1.08	0.59	1.01	1.14	1.46	1.84
**% decline in Anti-PF4 IgG antibody from diagnosis to latest follow-up**	38.2%	58.1%	69%	-9.8%	51.1%	37.5%	20.7%
**Timing of latest follow-up (days post vaccine)**	200	277	280	217	158	200	203

CVST, cerebral venous sinus thrombosis.

Supplementary Table 1: Summary of Clinical Characteristics of Patients with VITT.
